# Concomitant Cytomegalovirus Viraemia in Multisystem Inflammatory Syndrome in Adults (MIS-A) following COVID-19

**DOI:** 10.1155/2024/8852063

**Published:** 2024-04-01

**Authors:** Benjamin Valente-Acosta, Francisco Moreno-Sánchez, Yvette Neme-Yunes, Sara López-Rueda, Irma Hoyo-Ulloa, Mónica Garcia-Gutierrez

**Affiliations:** The American British Cowdray Medical Center, Sur 136 116, Alvaro Obregon, Mexico City 01120, Mexico

## Abstract

Multisystem inflammatory syndrome in adults (MIS-A) is recognised as an infrequent complication of coronavirus disease 2019 (COVID-19). This syndrome occurs following COVID-19 infection in some individuals and is characterised by inflammation of multiple organ systems, such as the heart, liver, bowel, and lymph nodes. Cytomegalovirus (CMV) viraemia is associated primarily with immunosuppression. In COVID-19 patients, it has been reported in severe and critical cases. We present a case of an adult patient diagnosed with MIS-A and concomitant CMV viraemia.

## 1. Background

Multisystem inflammatory syndrome in adults (MIS-A) is a rare hyperinflammatory syndrome that was initially described in children (MIS-C) previously infected with severe acute respiratory syndrome coronavirus 2 (SARS-CoV-2) [1]. MIS-C is a post-infectious inflammatory condition associated with an abnormal immune response, cardiac dysfunction, and multiorgan involvement. This condition is more commonly observed in children aged 5–13 years. The diagnosis is clinically based on the following criteria: age <21 years, clinical severity necessitating hospitalization, absence of an alternative diagnosis, fever, C-reactive protein >3 mg/dL, prior documented SARS-CoV-2 infection by detection of RNA, antigen, or antibodies, and multisystem involvement in at least 2 categories, including cardiac involvement, mucocutaneous involvement, shock, gastrointestinal involvement, and haematologic involvement (thrombocytopenia or lymphopenia) [2].

Similar to children, MIS-A induces multiorgan inflammation in adults and manifests with fever, abdominal pain, vomiting, diarrhoea, rash, and nonpurulent conjunctivitis [3]. MIS-A could manifest as severe symptoms including myocardial dysfunction, thrombosis, hypotension, and organ failure. The pathophysiology of the disease is not fully elucidated, though multiple immunologic mechanisms are proposed to be involved, such as molecular mimicry, epitope spreading, and bystander activation [4]. Cytomegalovirus (CMV) viraemia has been described in patients with critical conditions or who are immunosuppressed. Therefore, it is recognised as a proxy of immunosuppression. Also, CMV viraemia has been described in severe coronavirus disease 2019 (COVID-19) cases [5]. Specific organ affection has been described, especially in the lung, as CMV pneumonitis [6]. In multisystem inflammatory syndrome, CMV has been described only in children [7]. Here, we present a case of a man in his 40s with MIS-A and concomitant CMV viraemia.

## 2. Case Presentation

A Hispanic man in his 40s presented to the emergency department with a 10-day history of fever and malaise. One month prior, the patient had a mild COVID-19 infection that was successfully treated symptomatically. He had a relevant history of recently being diagnosed with hypertension and recent travels to Sweden and a ranch in Valle de Bravo, Mexico.

Five days prior to the first consult in the emergency department, the patient was evaluated by another physician. At that time, the patient complained of fever, headache, photophobia, chills, and diarrhoea. The physician prescribed ceftriaxone 1 g intramuscular every 24 horas and ordered blood tests, which revealed mild thrombocytopenia (146,000 per mm^3^), elevated liver enzymes (AST: 97 U/L, ALT: 91 U/L, GGT: 107 U/L, DHL: 319 U/L), and high C-reactive protein (CRP) levels (2.9 mg/dL). The viral hepatitis panel was negative for active infection, and the Venereal Diseases Research Laboratory (VDRL) test and HIV test were negative. CMV was positive for only immunoglobulin G (IgG), indicating prior infection. A liver ultrasound was performed, and its results showed fatty infiltration.

Despite the previous treatment, the patient continued experiencing intermittent fever, myalgias, arthralgias, headache, and abdominal pain, necessitating his admission to our emergency department. At the time, the patient had a fever of 39°C, heart rate of 120 bpm, and tissue oxygenation saturation (StO_2_) of 89%. A nonpurulent conjunctivitis and a maculopapular rash were observed on the abdomen and the anterior thorax. The abdomen was tender but showed no signs of peritoneal irritation. There were no palpable cervical or inguinal lymphadenopathies, and the rest of the physical examination was unremarkable.

In the emergency department, an extensive work-up for the cause of the fever was conducted by our infectious disease team, which then requested laboratory and imaging studies. Given the clinical picture and the unknown origin of the fever, a mononucleosis-like syndrome was first diagnosed, and the patient was admitted for further management and diagnostic work-up. Upon admission, antibiotic treatment was continued with ceftriaxone 1 g intravenous every 12 hours, and doxycycline 100 mg every 12 hours by mouth was added to the antibiotic's regimen, given the patient's history of recent travels to a ranch.

Initial evaluation included serology for the Epstein–Barr virus (EBV), toxoplasma, rubeola, CMV, herpes simplex virus 1 and 2 (HSV), human immunodeficiency virus (HIV), Coxiella, hepatitis A, B, C, and E virus, Leptospira, Bartonella, Ehrlichia, Anaplasma, dengue, chikungunya, Zika, and Brucella, which were negative for recent or current infection. Multiplex polymerase chain reaction (PCR) tests for respiratory and gastrointestinal pathogens were negative, including the PCR test for SARS-CoV-2. Blood and urine cultures were also negative.

Blood tests revealed a normal complete blood count, although toxic neutrophil granulation and activated atypical lymphocytes were observed on the thin film. Remarkably, the results of the liver function tests were progressively worse (Table 1, Biochemical biomarkers). Elevated levels of D-dimer (2974 ng/ml), ferritin (944 ng/ml), beta-2-microglobulin (5.769 mg/L), and IL-6 (11.2 pg/mL) were observed. The test results for antinuclear antibodies, anti-DNAds, anti-SSA, anti-SSB, antismooth muscle antibodies, anticitrullinated protein antibody, p-ANCA, and c-ANCA were all negative. Rheumatoid factor was slightly positive at 17.8 U/ml (reference values: 0–14 U/ml). C3 and C4 protein levels were normal. Test results for SARS-CoV2 IgG antinucleocapsid antibody were positive, indicating prior infection. An initial chest and abdominal computed tomography (CT) scan showed splenomegaly and retroperitoneal lymphadenopathies. The echocardiogram was normal, with preserved ejection fraction.

The patient's condition persisted during the hospital stay, with daily fever, intense body pain, and malaise despite paracetamol; however, the diarrhoea stopped. Because of these outcomes and the patient's clinical picture, haematologic neoplasia was suspected at this point. The haematology unit ordered free serum kappa and lambda chains, immunofixation, and protein electrophoresis. The protein electrophoresis detected polyclonal hypergammaglobulinemia. Peripheral blood immunophenotyping did not detect any cellular clones but revealed an elevated T lymphocyte subpopulation (CD3/CD8+).

**Figure 1:**
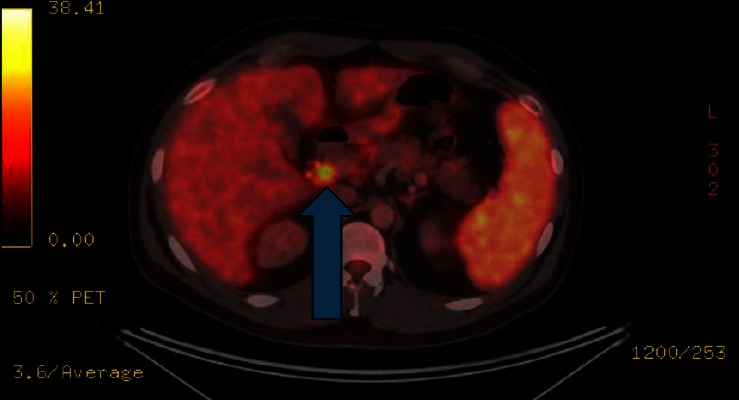
PET scan showing infradiaphragmatic lymphadenopathies and retroperitoneal intercavoaortic lymphadenopathies, measuring 12 × 10 mm, maximum SUV of 9.8.

A 18F-fluorodeoxyglucose (18-F) positron emission tomography scan (PET-CT) was requested, which revealed hypermetabolic retroperitoneal and intercavoaortic lymph nodes, measuring 12 × 10 mm, with a maximum standardized uptake value (SUV) of 9.8, inversion of liver/spleen metabolism, and hepatosplenomegaly (Figure 1). Furthermore, HIV, human herpesvirus-8 (HHV-8), CMV, and EBV load tests were requested, and the results of the latter two were positive. The EBV viral load test result was positive but below the threshold for quantitative detection (<124 UI/ml). The CMV viral load was 3,933 UI/ml, and CMV IgG was positive, indicating CMV reactivation in the context of an immunocompetent patient.

Given the lack of a definite diagnosis and the suspicion of neoplasia, a retroduodenal lymph node biopsy and a liver biopsy were performed on the fifth day after admission.

While awaiting the biopsy results, the patient continued with fever. A follow-up CMV viral load test was requested two days after surgery, and it revealed an elevation in the CMV viral load (6,389 UI/ml; prior: 3,933 UI/ml). Therefore, antiviral treatment with ganciclovir (5 mg/kg intravenously) was initiated, although no organ damage was detected.

After one week from the surgery, the patient continued with daily fever; thus, methylprednisolone (80 mg per day) was prescribed. Despite corticoid treatment, the fever persisted, and on the twelfth day of admission, we decided to switch to high-dose dexamethasone (40 mg per day), and a new CT scan and bloodwork were ordered to re-evaluate the cause of the fever. The bloodwork results continued to report elevated liver enzymes.

**Figure 2:**
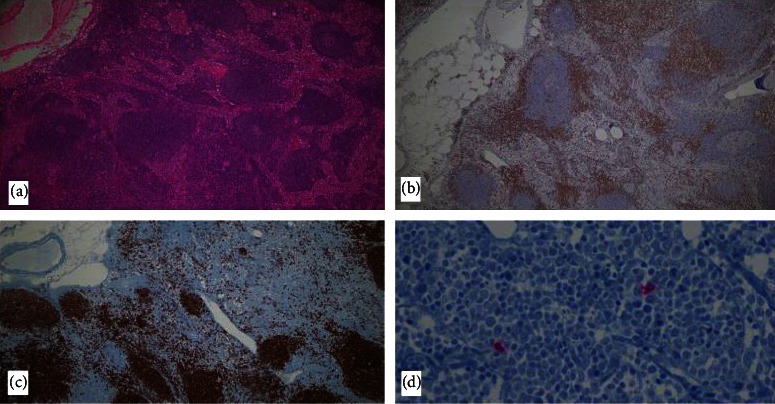
Lymph node biopsies: (a) hematoxylin and eosin 5x; (b) CD20, highlighting the T cell lymphoid component; (c) CD3, highlighting the B cell lymphoid component; (d) SARS-CoV-2 positive cytoplasmic in macrophages (in red).

The retroduodenal lymph node biopsy revealed sinusoidal and paracortical hyperplasia with small and medium lymphocytes, apoptotic cells, histocytes, plasmacytoid dendritic cells (CD123+), and hemophagocytes, with a few macrophages positive for SARS-CoV-2 in the cytoplasm (Figure 2). Liver biopsy showed a chronic inflammatory infiltrate negative for neoplasia. Immunohistochemistry was positive for CD20 and CD30 in reactive B lymphocytes; CD3 in reactive T lymphocytes; ki67 in 40% of the cells; Bcl-2 in plasmacytoid dendritic cells, kappa and lambda; and CD138 in plasmatic cells. Immunohistochemistry was negative for CMV.

Considering the patient's clinical evolution, adult multisystem inflammatory syndrome secondary to SARS-CoV2 and CMV reactivation were diagnosed. The patient meets the Centers for Disease Control and Prevention (CDC) case definition of MSI-A: age of 21 years or older; documented fever of above 38°C; exanthema and nonpurulent conjunctivitis; abdominal pain; vomiting or diarrhoea; thrombocytopenia; elevated CRP, ferritin and IL-6; and recent SARS-Cov2 infection [1].

After the initiation of treatment with high-dose dexamethasone on the twelfth day of hospitalization, the patient experienced resolution of the fever and improvement in liver function, as indicated by the test results. At hospital discharge, a dose-reduction regimen of dexamethasone was prescribed and valganciclovir 900 mg twice a day (oral ganciclovir prodrug) was continued to complete its 21-day scheme that was initiated with ganciclovir. During subsequent outpatient visits, weekly clinical exams and blood tests revealed further improvement in liver enzymes and fever resolution. A month after discharge, we stopped corticoid treatment after successful tapering. Furthermore, laboratory test results showed normal levels of D-dimer, LDH, CRP and liver enzymes.

## 3. Discussion

We present the case of a young man with controlled hypertension who presented with gastrointestinal symptoms, persistent fever, elevated inflammatory markers, altered liver function, and altered liver enzymes a month after a mild SARS-CoV-2 infection. His symptoms were consistent with MIS-A after an extensive diagnostic work-up eliminated other aetiologies.

MIS-A is a post-infectious hyperinflammatory syndrome that occurs after a SARS-CoV2 infection or COVID-19 vaccination [2]. It was first described in children (tagged MIS-C) in April 2020 [3]. The pathophysiology is not fully understood; however, it is known that after recovery from the infection, there is a dysregulated host immune response that produces a hyperinflammatory state 3–12 weeks (average of 4 weeks) after the initial viral infection [4].

Although the MIS-A incidence rate is unknown, it is less frequent than MIS-C [5]. Most of the information on MIS-A has been generated from clinical cases and series of cases [6, 7]. MIS-A predominantly affects males between 30 and 50 years of age. The most common presenting symptoms are fever and gastrointestinal symptoms, as in our patient [8]. Cardiac involvement is frequent but is not universally present, as evidenced by a recent systematic review and as observed in our case, in which there was no cardiac involvement [4]. Several treatments have been proposed, including glucocorticoids [9]. Interestingly, our patient did not respond positively to an 80 mg/day methylprednisolone dose (equivalent to 15 mg of dexamethasone), but he responded positively after a higher dexamethasone dose (40 mg/day) was administered. High-dose corticoids have been prescribed as a treatment option in children [10].

Reactivation of CMV has been observed in patients with COVID-19, likely due to the immune activation caused by the virus as well as the immunosuppressive treatments administered to patients [1, 11–13]. To the best of our knowledge, there are no reports of MIS-A with CMV viraemia or reactivation. In contrast, CMV reactivation in a child with multisystem inflammatory syndrome (MIS-C) related to COVID-19 has been reported [6]. In our patient, it is possible that the CMV viraemia was an epiphenomenon since he did not develop a CMV-related organ disease.

The PET scan showed abdominal lymphadenopathies, as has been previously reported in other MIS-A cases [9, 14]. The lymph node biopsy findings were of particular interest, as we were able to demonstrate the persistence of SARS-CoV-2 in macrophages. Although we were not able to assess viral load in the tissue or determine its viability, the positive immunohistochemistry results suggest that there may be a persistent inflammatory stimulus that triggers the immune system, thereby contributing to the pathophysiology of MIS-A [11, 15]. Our finding is consistent with that of previous reported cases on MIS-C, which showed SARS-CoV-2 persistence [16].

The primary limitation of our case is the absence of molecular immunology studies to elucidate the underlying mechanisms related to the development of MIS-A following SARS-CoV-2 infection. Additionally, as a single case report, we lack information on whether other therapeutic modalities, such as immunomodulatory agents or targeted antiviral therapies, might be more effective in specific cases. Finally, the presence of concomitant CMV viraemia in our patient adds complexity to the clinical presentation and treatment response. However, the precise role of CMV reactivation in the pathogenesis of MIS-A remains unclear.

## 4. Conclusion

In conclusion, we report a case of MIS-A in a man with concomitant CMV viraemia, following a mild SARS-CoV-2 infection. Our case underscores the importance of considering MIS-A in adults presenting with fever, gastrointestinal symptoms, and elevated inflammatory markers, even in the absence of cardiac involvement. High-dose corticosteroids, such as dexamethasone, may be effective in managing MIS-A, as evidenced by the resolution of symptoms in our patient.

## Figures and Tables

**Table 1:** Biochemical biomarkers.

	Prior to admission	On admission	Day 2	Day 4	Day 6	Day 8	Day 10	Day 12	Hospital discharge	Reference values
Aspartate aminotransferase (AST)	97 U/L	176 U/L	171.7 U/L	151.4 U/L	141.5 U/L	164.3 U/L	166.6 U/L	123.1 U/L	80.1 U/L	0.0–42 U/L
Alanine aminotransferase (ALT)	91 U/L	167 U/L	175 U/L	139 U/L	131 U/L	151 U/L	160 U/L	133 U/L	99 U/L	0–41 U/L
Lactate dehydrogenase (LDH)	319 U/L	408 U/L	400 U/L	347 U/L	308 U/L	320 U/L	372 U/L	322 U/L	187 U/L	122–222 U/L
Gamma-glutamyl transpeptidase (GGT)		278 U/L	429 U/L	426 U/L	577 U/L	654 U/L	813 U/L	638 U/L	458 U/L	0–60 U/L
Reactive C protein	2.9 mg/dL	2.1 mg/dL	5.59 mg/dL	4.66 mg/dL	1.41 mg/dL	1.56 mg/dL	5.55 mg/dL	3.93 mg/dL	0.93 mg/dL	0.00–0.50 mg/dL
Beta-2 microglobulin	—	5.760 mg/L				5.020 mg/L	6.740 mg/L			0.800–2.200 mg/L
D-dimer		2,947 ng/mL FEU				2,121 ng/mL FEU	2,682 ng/mL FEU		5,255 ng/mL FEU	40–500 ng/mL FEU
Ferritin		944 ng/mL					1,362 ng/mL		1,392 ng/mL	30–400 ng/mL
CMV viral load			3,933 UI/mL	6,389 UI/mL	24,639 UI/mL		2,343 UI/mL			Nondetectable

## Data Availability

Data used to support the study are included in the article.
